# The EPIDIA4Kids protocol for a digital epidemiology study on brain functioning in children, based on a multimodality biometry tool running on an unmodified tablet

**DOI:** 10.3389/fpubh.2023.1185565

**Published:** 2023-05-30

**Authors:** Vanessa Douet Vannucci, Théo Marchand, Alexandre Hennequin, Hervé Caci, Pascal Staccini

**Affiliations:** ^1^R&D Lab, O-Kidia, Nice, France; ^2^URE Risk Epidemiology Territory INformatics Education and Health (URE RETINES), Université Côte d’Azur, Nice, France; ^3^Bioelectronic Lab, Ecole des Mines de Saint-Étienne, Gardanne, France; ^4^Hôpitaux Pédiatriques de Nice CHU Lenval, Nice, France; ^5^Centre de Recherche en Épidémiologie and Santé des Populations (CESP), INSERM U1018, Villejuif, France

**Keywords:** digital epidemiology, multidimensional assessment, biometry, child development, neurodevelopmental disorders, cognitive and behavioral performances

## Abstract

**Introduction:**

Neurodevelopment and related mental disorders (NDDs) are one of the most frequent disabilities among young people. They have complex clinical phenotypes often associated with transnosographic dimensions, such as emotion dysregulation and executive dysfunction, that lead to adverse impacts in personal, social, academic, and occupational functioning. Strong overlap exists then across NDDs phenotypes that are challenging for diagnosis and therapeutic intervention. Recently, digital epidemiology uses the rapidly growing data streams from various devices to advance our understanding of health’s and disorders’ dynamics, both in individuals and the general population, once coupled with computational science. An alternative transdiagnostic approach using digital epidemiology may thus better help understanding brain functioning and hereby NDDs in the general population.

**Objective:**

The EPIDIA4Kids study aims to propose and evaluate in children, a new transdiagnostic approach for brain functioning examination, combining AI-based multimodality biometry and clinical e-assessments on an unmodified tablet. We will examine this digital epidemiology approach in an ecological context through data-driven methods to characterize cognition, emotion, and behavior, and ultimately the potential of transdiagnostic models of NDDs for children in real-life practice.

**Methods and analysis:**

The EPIDIA4Kids is an uncontrolled open-label study. 786 participants will be recruited and enrolled if eligible: they are (1) aged 7 to 12 years and (2) are French speaker/reader; (3) have no severe intellectual deficiencies. Legal representative and children will complete online demographic, psychosocial and health assessments. During the same visit, children will perform additionally a paper/pencil neuro-assessments followed by a 30-min gamified assessment on a touch-screen tablet. Multi-stream data including questionnaires, video, audio, digit-tracking, will be collected, and the resulting multimodality biometrics will be generated using machine- and deep-learning algorithms. The trial will start in March 2023 and is expected to end by December 2024.

**Discussion:**

We hypothesize that the biometrics and digital biomarkers will be capable of detecting early onset symptoms of neurodevelopment compared to paper-based screening while as or more accessible in real-life practice.

## Introduction

Neurodevelopmental and related mental disorders are one of the most frequent disabilities among young people. As stated by OCDE (2022), more than 166 million youth worldwide are affected by such conditions that are of significant concern in term of public health but also of economic burden (1) and social welfare (2).

Neurodevelopmental disorders (NDDs) are defined by the Diagnostic and Statistical Manual 5th Edition [DSM-5-TR (3) and in the International Classification of Diseases 11th version (ICD-11) (4)], as all structural or/and brain functioning anomalies that occur during a child development. They encompass intellectual handicaps (intellectual disabilities), attentional deficit/hyperactivity disorder (ADHD), communication disorders (language, speech, or communication deficits), specific learning disabilities (SLD, difficulty reading, or with written expression, or with numbers and mathematical reasoning etc.), developmental coordination disorder (DCD or dyspraxia), autism spectrum disorder (ASD). NDDs occur from early childhood and persist to adulthood for 50% (5). Negative impacts such as school dropout, loss of self-esteem or social interaction (6), severe neglect at home or bullying at school, can and do have enduring and damaging effects on the development of core cognitive and emotional skills, that are often lifelong conditions (1, 7).

The pathophysiology of NDDs is complex and remains largely unknown although multiple pre- and peri-natal environmental factors along with polygenic influences have been recognized (8–10). They are often associated with other somatic or psychiatric diagnoses (comorbid, concomitant or in continuum) (11–15). However, there are no psychological or biological consensual assessments, nor imaging techniques to make ascertain diagnosis of NDD, which today relies mainly on clinical evaluation. Furthermore, strong overlaps across NDD phenotypes make both group and distinction of each disorder difficult in real-life practice.

In addition to the clinical complexity, significant barriers prevent from NDDs and disclosing concerns. These include the stigma of mental health (16), lack of understanding of whether symptoms are abnormal or a typical experience, lack of time and lack of accurate trustable assessment tools (17), and knowledge of their interpretation, lack of referral processes and options, having providers or support persons underestimate their symptoms and concerns, and fear that reporting symptoms will lead others to think that parents or relatives are incompetent in their parenting or educating role (7, 18), leading to severely under detected and undertreated children with an estimated prevalence of NDDs quite high without standardized screening (19–21). Routine, standardized screening would significantly improve detection of neurodevelopmental alterations. However, the scarcity of human health resources poses a major deterrent to routine screening (7). E-screening has the potential to increase efficiency of mental healthcare by re-allocating limited human resources where they are most needed—in-depth follow-up assessment, referral, and treatment (22). It is a low-resource option that can be embedded in current routine practice across various settings and providers (for example, family physicians, child psychiatrists, psychologists, speech therapists, nurses) and thus will increase access to routine screening.

Importantly, e-screening through a careful gamification can address the most prominent barriers to screening identified by children, relatives, and healthcare providers. It would also ease the acceptability and feasibility in real life practice and provide personalization to patient needs (23) with real-time data (24–27) along anonymity (28–30). Recently, digital biomarkers have shown a strong potential to predict mental issues in adults (31–33), but none has investigated in children whether biometry could be relevant for a better understanding of neurodevelopmental conditions and for the identification potential subtypes as a NDD continuum in children (34). To our knowledge, no studies have evaluated e-screening based on multimodality biometry and digital biomarkers in children and in ecological context.

There is a clear need for a rigorous evaluation of the feasibility, acceptability, and psychometric performance of e-screening for large scale children and adolescent cohort. A key consideration for evaluation is to determine whether gamified e-screening delivered through an unmodified tablet are valid and reliable for use compared to well-established assessment tools. For example, it has been shown that some tools have different psychometric properties when delivered online, suggesting a need for different cutoff points (35).

Taken together, the need to identify screening and assessment tools that are acceptable to both children and health professionals, overcome barriers to accessibility-to-care, to implementation into routine care, and are cost-effective and clinically useful, is crucially needed.

Today, billions of people are increasingly generating and collecting health-related data online (36) or on mobile devices. The opportunities for more accurate and effective health services are enormous (37, 38). Digital epidemiology uses the new and rapidly expanding digital data streams from mobile devices to advance public and personalized health (39, 40). These datasets are then used by artificial intelligence algorithms to improve our understanding of health and disorders dynamics, both in individuals and in the general population. In natural environment, digital epidemiology represents a new approach that aims at measuring human behavior and may, combined with clinical (endo)phenotypes, enhance capability and sensitivity in early identification, diagnosis, and management of health conditions. Moreover, such a combined approach may easily allow clinicians to perform a more personalized and patient-tailored diagnostic and therapeutic approach.

As children and adolescents are so familiar with digital technologies (41, 42), building the right services specifically designed and dedicated to them will help to prevent, monitor, and intervene more appropriately on neurodevelopmental and related mental disorders at a larger scale.

Here, we investigate how digital phenotyping integrated with clinical (endo)phenotypes constitutes a new method for building comprehensive datasets based on digital epidemiology to create meaningful metrics of cognition, emotions, and behavior specific to children and adolescents. These detectable and measurable features will help understanding better brain development and hereby identifying prominent predictive variables to achieve dimensionality reduction and risk factors for NDDs.

**Purpose** (Table 1)—the EPIDAI4Kids study will examine brain functioning in children aged 7 to 12 years using biometrics and digital biomarkers to create meaningful metrics of cognition, emotions, and behavior specific to children and adolescents, while they are playing at gamified assessments on an unmodified tablet.

**Table 1:** Primary and secondary objectives, research questions, and hypotheses.

Primary objective	Research question	Outcome	Measures	Testable hypothesis
To create and compare a normative base of brain functioning in children aged 7 to 12 years using multimodality biometry e-measurements vs. paper-based screening	Is the neuro-developmental e-assessments as or more acceptable to children and health practitioners than paper-based screening?	Acceptability: % children in the study failing finishing the e-assessments	Acceptability	Higher proportion of children and health practitioners will affirmatively respond to acceptability for the e-measurement
		% of participants reporting that e-questions and psychometric games are easy to navigate around on the tablet	Qualitative; semi-structured interview	
			Quantitative: proportion of participants feeling uncomfortable	
To compare psychometric properties (sensitivity, specificity, cutoff points) of paper-based screening vs. the psychometric games?	What are the performances in cognition, emotion and/or behavior in children aged 7 to 12 years on child neurodevelopment?	Efficiency: correlations between scoring of paper-based screening performances and biometrics within each participant and inter-examinator	Quantitative: video and audio will be recorded for about 30 min while the children are playing on a unmodified tablet. Eye-, face- digit- and sound- will be extracted from recorded video by ML/DL algorithms.	Strong correlations between paper-based screening performances (psychometric properties) and multimodality biometrics within each participant.
				Less variability inter-examinator on e-assessments than on paper-based screening
Secondary objective	Research question	Outcome	Measures	Testable hypothesis
To compare feasibility of the multimodality biometry e-measurements?	Is the neuro-developmental e-assessments as or more feasible to children and health practitioners than paper-based screening?	Feasibility: % children in the study failing finishing the e-assessments	Qualitative: semi-structured interviews	Higher proportion of children and health practitioners will affirmatively respond to feasibility for the e-measurement
		% of health practitioners reporting that e-questionnaires and psychometric games are easy to navigate around on the tablet		
To validate the clinical relevance of multimodality biometrics	Are multimodal biometry e-measurements clinically relevant metrics for neurodevelopmental and mental health?	Match mobile EEG biomarkers for neurodevelopmental and mental health with multimodality biometrics	Quantitative: electroencephalographic (EEG) biomarkers of cognitive, emotional, and behavioral performance in children aged 7–12 years	Compare EEG signals obtained while children are playing with biometrics measurements related to well-characterized EEG signatures for related mental health in children (P2, N170, P300 amplitude and latence, Ratio Theta/Beta (TBR), Frontal Theta and Delta Power, Posterior Alpha et beta Power)
To determine factors associated with level of disclosure of symptoms related to neurodevelopmental and mental health	What factors are associated with risk of neurodevelopmental and mental health?	Identification of factors that significantly increase odds of neurodevelopmental and mental health reported symptoms	Quantitative: (1) demographic variables (age, gender, SES), (2) medical status (current NDD status, history), (3) psychopathological scores and quality of life (BRIEF, CBCL, ADHD scale, ASQ-10, PSQI, Grit questionnaire, SMQ)	Compared to paper-based screening, e-screening promotes greater disclosure
To determine multimodality biometric e-assessment as a monitoring tool		Examination of significant changes of either or both scores compared with baseline (repeated testing)	Quantitative: compared 4-month follow-up scores of e-assessment and paper-based screening with those obtained at baseline	More variability of scoring in those obtained from paper-based screening will be expected than those from e-assessment

### Primary objective

The primary objective of the study is to determine whether a normative base relying on multimodality biometry e-measurements tool, called XAI4Kids®, can evaluate brain functioning, i.e., the psychometric properties (sensitivity, specificity, reliability, reproducibility, and positive/negative predictive values) in children aged 7 to 12 years and evaluate the efficiency and acceptability of such e-screening tool compared to various current convergent validity measures of brain functioning (Figure 2).

#### The multimodality biometry tool description

The XAI4Kids® Multimodality Biometry tool has been developed by O-Kidia, SAS, and is composed of several technological components (Tables 2 3):

▪ The unmodified tablet: data will be collected in ecological context during the session to minimize stigma. Participant’s main biometry parameters (e.g., eye-, face-, and digit-tracking, frequencies, etc.), clicking, taping, and video from the intrinsic camera will be recorded to reflect of the child’s performances.
▪ The O-Games: a series of gamified psychometric sessions, which will engage the participants to perform as the best, and stimulate the cognitive, emotional, and behavioral functions of each participant. Games were developed for Android with Unity^1^ and will be installed on each participant’s table.

**Table 2:** Study workflow at baseline.

Name	Description	Time	Who	Visit
Screening worksheet	Eligibility criteria	10 min	Y, P	B
Modified Ohio State TBI Screen-Short Ver	TBI rule-outs at BL; TBI	5 min	P	B
Demographics domain	demographics, race, gender, family structure, SES	20 min	P	B
Medications	Medical history, medications	5 min	P	B
Pittsburgh Sleep Quality Index	Sleep habits	5 min	P	B, T2-end
Children’s Sleep Habits Questionnaire	Sleep habits	5 min	Y	B, T2-end
Questionnaires for parent	ADHD-rating scale, Children Behavior Check, BRIEF, DCDQ, Conners 3	20 min	P	B, T2-end
Questionnaires for child	ADHD-rating scale, ASQ-scale, Grit scale, and SMQ	20 min	Y	B, T2-end
WISC-V (substest)	7 subtests Symbol, Cubes, Code, Comprehension, Letter-Number sequences, matrix, and similitude	45 min	Y	B, T2-end
O-Games battery	5 psychometric games	20–35 min	Y	B, T2-end
Breaks	As much as needed	20 min	Y	B, T2-end

**Table 3:** Convergent validity measures for the XAI4Kids^®^ e-measurement tool.

What it measures	Stream data type	Convergent validity measures
Visio-spatial processing	Rocket	WISC-V, block design
	SES4ME	WISC-V, symbol search
	O-TOM	WISC-V, barrage
Working memory	Untangle, EEG (P3b)	WISC-V, letter-number sequences
Episodic memory	O-G	WISC-V, letter-number sequences, digit-span
Attention	Rocket, connect	WISC-V, digit span, coding, symbol search
Speed processing	Connect	WISC-V, symbol search (immediate recall) coding
Language	O-KIDO	WISC-V, comprehension, vocabulary,
Executive functions	O-MAZE	WISC-V, BRIEF, vocabulary,
Auditive Attention	O-METRO	WISC-V, digit span
Anxiety	EEG and video-analysis	CBCL subtest
Sleep	EEG and video-analysis	PSQI
Motivation	EEG and video-analysis	SMQ, grit scale
Emotional control	EEG and video-analysis	BRIEF
Social abilities	O-TOM, EEG, and video-analysis	CBCL subtest, ASQ
Visual motricity	Eye-tracking	WISC-V, symbol search, coding
Visual acuity	Eye-tracking	WISC-V, symbol search

In addition, the tool is constituted by:

▪ The AI module: the backend part of XAI4Kids® system will allow the clinical recommendations of biometry extracted features (43–48) based on machine and deep learning algorithms. Data-driven coefficients from first-level analyses for each participant will be provided for each analysis. To facilitate Big Data deployment, and re-use, the computational workflows will be implemented as virtual machines (VMs), deployable on multiple computing resource and on local workstations. Each metric will be obtained from time-series data and stored in the time-series analysis database. The query object graph analysis method will be used to enable analysis sharing among analysts by preventing duplicate processing and data explosion (49).
▪ The artificial setting: to extract features of interest from the signals, tracking software and algorithms have been specially developed for feature extractions.

### Secondary objectives

The analysis of the impact on the patients’ functional status as measured by EEG, socioeconomical and psychosocial markers, and quality of life, will be considered as secondary outcomes.

Four secondary objectives are (i) Describe the subgroup profiles as a function of NDDs related symptoms intensity, potential comorbidities and neuropsychological performances; (ii) Compare feasibility of the multimodality biometry e-measurements vs. current measurements of neurodevelopment; (iii) Determine factors associated with level of disclosure of symptoms related to neurodevelopmental and mental health; (iv) Validate the clinical relevance of multimodality biometrics using mobile electroencephalography (EEG); (v) Determine multimodality biometric e-assessment as a monitoring tool.

### Research questions and hypotheses

Combining cognition, emotion, behavioral, and brain measurements at once through an unmodified tablet is a novel approach that could facilitate NDD evaluation in real-life practice at large scale. Overall, we hypothesize that brain functioning e-screening with XAI4Kids® in real life practice, will be as or more feasible and capable of capturing the heterogeneity of NDDs in the population, and ultimately serve as a first instance transdiagnostic evaluation for NDDs.

## Methods

The study was designed to examine whether multimodality biometry markers can reflect brain functioning as measured by cognitive, emotional, and/or behavioral performances in children aged 7 to 12 years using an unmodified tablet.

### Ethics approval

The EPIDIA4Kids study was approved by the Committee for the Protection of Individuals Sud-Est II (National French Register under the number 2022-A00766-37), the Commission Nationale de l’Informatique et des Libertés (CNIL) and was registered in October 2022 on ClinicalTrials.gov under the number (NCT05577533).

### Study design

**Figure 1:**
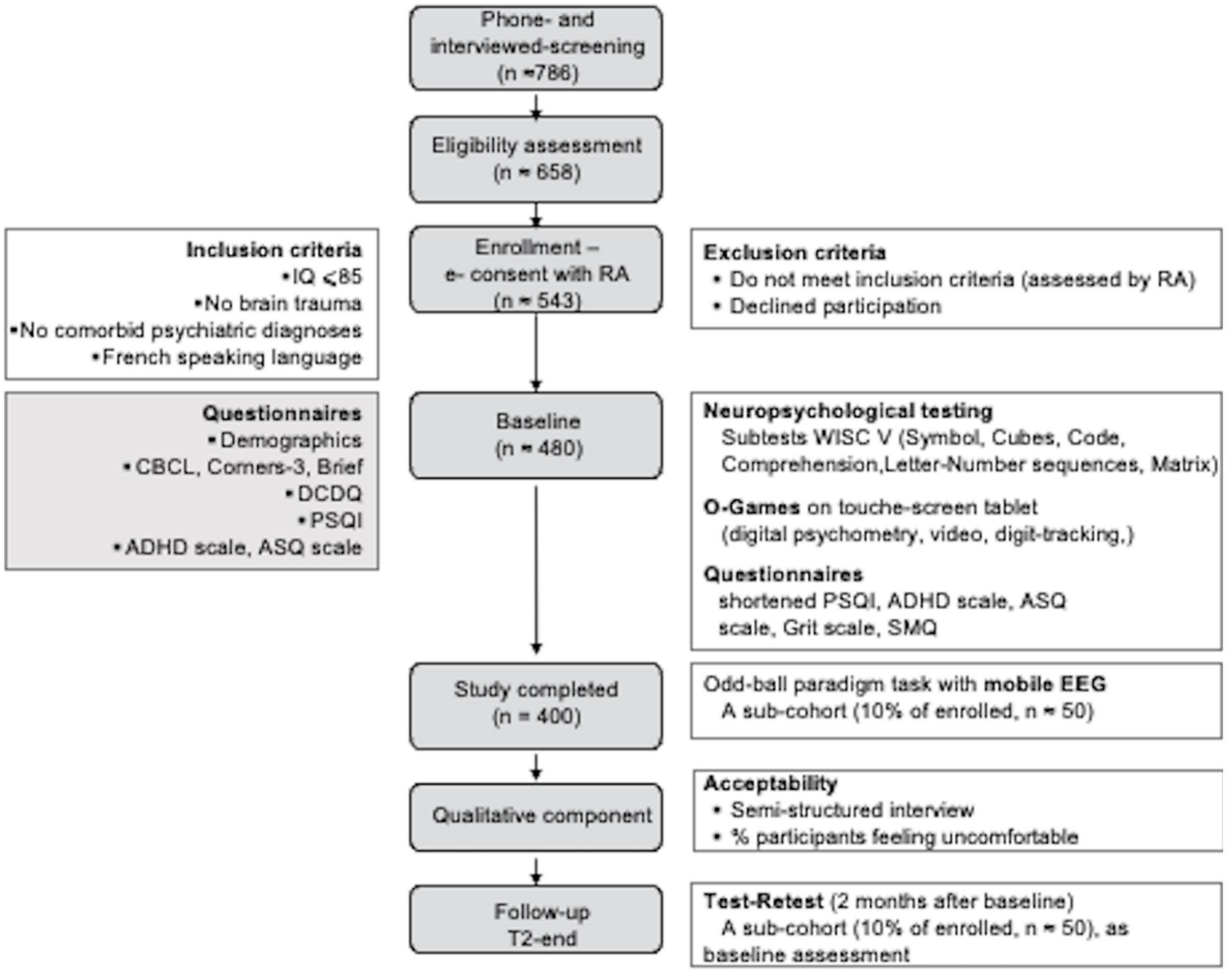
EPIDIA4Kids flow diagram. We plan to screen ≈ 786 individuals initially by telephone and 658 of these potentially eligible participants in person, to identify ≈ 543 participants who will fulfill our study criteria and 400 completing the baseline evaluation. We expect that only ≈ 500 will return for the 2-month follow-up. Questionnaires, interview, and testing will be performed by legal representative (gray boxes) and children (open boxes). IQ, Intellectual quotient; RA, research assistant; CBCL, Child Behavior Checklist; BRIEF, Behavior Rating Inventory of Executive Function; DCDQ, Developmental Coordination Disorder Questionnaire; PSQI, Pittsburg Sleeping Questionnaire Inventory; ADHD, Attention Deficit Hyperactivity Disorder; ASQ, Autism Spectrum Questionnaire; WISC-V, Wechsler Intelligence Scale for Children; SMQ, School Motivation Questionnaire; EEG, Electroencephalogram.

EPIDIA4Kids is an observational uncontrolled multi-center study (several cities in France) during which no treatment or placebo will be administered. Complementing children’s assessment at baseline (T0), a subsample of 10% of the participants will be re-assessed after 2 months (end-T2). At least one of the parent or legal representatives and children will also answer questionnaires and will be interviewed using a semi-structured instrument (Figure 1).

### Aims of the study

The primary aim is to create a brain functioning normative database relying on digital epidemiology in children aged 7 to 12 years using multimodality biometry. Brain functioning will be analyzed as variations of three dimensions related to cognition, emotion, behavior, and their respective deviation related to NDDs symptoms (Table 1).

The secondary aims are defined as following:

▪ To identify subgroup profiles as a function of (1) NDDs related symptoms intensity, (2) pattern of psychiatric comorbidities, (3) neuropsychological results.
▪ To compare feasibility of the multimodality biometry e-measurements to current screening measurements.
▪ To validate the clinical relevance of multimodality biometrics using mobile electroencephalography (EEG).
▪ To determine factors associated with level of disclosure of symptoms related to neurodevelopmental and mental health.
▪ To determine multimodality biometric e-assessment as a monitoring tool.

### Participant

The inclusion criteria will be: (i) boys or girls of any ethnicity, aged 7–12 years (typically developing and diagnosed with NDDs); (ii) French native speakers; (iii) normal hearings, normal or corrected-normal vision; (iv) no brain trauma; (v) an estimated intelligence quotient of 85 and above (exclusion if score < 25th percentile) based on Weschler’s subtests Matrix Reasoning and Similarities (50); (vi) no history of co-morbid psychiatric illness that might confound the analysis of the study (e.g., schizophrenia, obsessive-compulsive disorder, major depression, or bipolar disorder); (vii) ADHD medications and Selective serotonin reuptake inhibitors (SSRIs) are not exclusionary since use of these medications is associated with neurodevelopmental disorder condition, but the treatment will be suspended 2 days before any cognitive evaluation.

The exclusion criteria will be: (i) failure to meet the inclusion criteria; (ii) lack of written informed consent: the child and at least one of the parents (or the legal representative) must sign the informed consent form.

### Recruitment

Recruitment procedures will be similar across sites, and trained staff will use a standardized script to determine eligibility in study participation. Participants (child and parent/legal representative) will be recruited from the local community, outpatient’s clinics, or referrals from physicians by advertising or interception method. They will complete the consent electronically and receive the automatically generated copy via email (Figure 1). The research assistants will be available to answer questions about study participation and tablet logistics. EPIDIA4Kids will ensure full time recruitment, to include a larger number of participants, as well as participants from a variety of social, geographic, and ethnic backgrounds, thereby increasing the accuracy and “generalizability” of the data collected as approved by the Comité de Protection des Personnes (CPP). Recruitment will be monitored across sites and, as recruitment proceeds, adjustments will be made to ensure that sociodemographic targets are reached. Once the compliance with the inclusion and exclusion criteria of the study is verified, the informed consent will be obtained, and baseline evaluation will be processed. The study will start in March 2023 and is expected to end in December 2024. Four hundred participants are targeted to complete the study.

**Figure 2:**
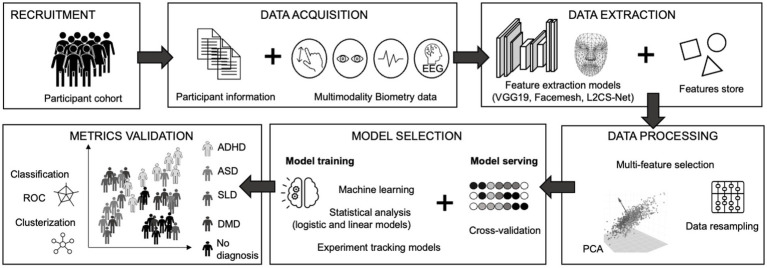
The EPIDIA4kIDS proposed framework. We will collect data from varied sources, including participant information with questionnaires and semi-structured interviews regarding their background, demographics, and medical history. Using unmodified tablet, we will gather data from video, digit-tracking, and EEG (for a sub cohort) as time-series logs. Based on various performance metrics, relevant features will be extracted automatically using various machine and deep learning algorithms to create a store of features clinically relevant based on the literature. Relevant features will be then processed and selected through statistical analysis and model selection that combined model training and model serving. Afterwards, analysis is performed to validate the relevance of the selected metrics for identification of cognitive, behavioral, and emotional states related to NDDs. EEG, Electroencephalogram; PCA, Principal Component Analysis; ADHD, Attention Deficit Hyperactivity Disorder; ASQ, Autism Spectrum Disorder; SLD, Specific Learning Disorders; DMD, Developmental Motricity Disorder; ROC, Receiver Operating Characteristic.

### Questionnaires and neuro-assessments

All questionnaires and neuro-assessments are summarized in Table 4. At baseline and T2-end, the visit will be conducted in a sequential manner with breaks. First, children will perform neuropsychological assessments through digitalized or paper/pencil WISC-V battery [1.5 h, (50)] with the Similarities, Vocabulary, Coding, Symbol search, Block design, Digit, Matrix reasoning subtests. Secondly, questionnaires will be answered digitally for:

▪ Demographic information (age, gender, language spoken, family structure, school, previous diagnosis for neurodevelopmental disorder) and scoring of manual laterality [Edinburgh handedness, short version, 4 items (54), estimated intellectual quotient (50), and sleep quality (Pittsburgh Sleep Quality Index, 11 items) (55)].
▪ The French version of the ADHD-Rating Scale (56) incorporating the 18 criteria rating Inattention (sum of odd items scores) and Hyperactivity-Impulsivity (sum of even items scores) symptoms.
▪ The French version of the Children Behavior Check List [short version, 32 items, (57) and Conners-3 (58)—psychopathological behavioral or emotional traits].
▪ The Autism Quotient List French version scoring [10 items, (59)]—psychopathological behavioral or emotional traits.
▪ The Developmental Coordination Disorder Questionnaire [DCDQ, 15 items, (60)].
▪ The Behavioral assessment of executive function scoring [BRIEF, 72 items, (61)].
▪ The French versions of the School Motivation Questionnaire [SMQ, 44 items, (62)] and the Grit-Scale for Children and Adult [GSCA, 12 items, (63)].

**Table 4:** Explanatory variables for the XAI4Kids^®^ e-measurement tool.

Neuropsychologic tests	Variables
	Reaction time: average reaction time (RT) for every executed task
	Hits: number of correct answers
	Total errors: total number of errors
	Wrong answers: number of answers that are not omission, perseverance and outliers
	Omission: reaction time = 0 ms
	Distractors: number of answers looking similar to the target answer
	Persévérance: 0 ms < reaction time < 100 ms
	Outliers: standard deviation of reaction time (exécution) < 3 x standard deviation (si RT≠0)
	Hits RT: median of reaction time for correct answers
	Hits RT SE: standard error de Hits RT
	SE variability: standard deviation of the 3 standard errors calculated for each task block (mesure of the participant variability)
	Hits rt block change: slope change for RT between blocks (vigilance)
	Hits SE block change: slope change for standard errors of RT between blocks (consistency and vigilance measure)
	Hits correct answers: Combination order of correct answers (behavioural trajectory measure)
	Hits correct answers through realization time: Combination order of correct answers (behavioural trajectory measure) by reaction time (speed processing measure)
	Distance deviation to stimuli: graphological fine motor skills
	Emotion analysis after stimuli: emotional control
	Attentional network: gap between medians of reaction time when the answer is correct or when it is wrong.
	Alerting: RTsans cue – RTdouble cue
	Orienting: RTcue centrale – RTorienting cue
	Conflict: RTflanker incongruent – RTflanker congruent
Video-tracking	Emotion, attention, social interaction: postural stability (51)
	Attention, information processing (semantic access, learning disability): fixation (amplitude, direction, interval time)
Digit-tracking	Attention, motricity: contact point (52)
	Real-time track (exploration time, direction, cumulative distance)
Eye-tracking	Attention, coordination: pro/anti-saccades (amplitude, direction, reaction time) (53)
	Attention, social interaction, information processing (semantic access, learning disability): fixation (amplitude, direction, interval time)
	Attention/stress: blink rate, pupil size, near point convergence

### Electro-encephalographic measures

EEG signals will be recorded on 50 children randomly chosen among the participants while they will perform the gamified psychometric tasks (O-Games). EPOC^X^, a 14-electrode wearable device (Emotiv, San Francisco) will be used to this extent, and data will be filtered using EmotivPRO+ version 2.7.2 (Emotiv). Large artifacts (such as eye and muscle movements, and heart noise), identifiable as outliers in each EEG, will be excluded. Independent Components Analysis will be run in MATLAB (MathWorks, Natick, MA) to identify N1, P1, N2, P2, and P3 waves amplitudes and latencies.

For pre-processing and analyzing, Neuroscan EEG data will be downsampled to 128 Hz to match the sampling rate of the EPOC^X^ system. EEG data will be filtered from 0.1 to 30 Hz for the sample of frequencies. The system will be used to acquire and analyze ERPs in children Epochs obtained with EPOC^X^ and with Neuroscan will be compared, averaged and substracted to produce a mismatch negativity (MMN) waveform. The ERPs produced using the two different devices will be analyzed in different ways. Firstly, the total number of accepted epochs used to compare the quality of the Neuroscan and EPOC^X^ collected data. Second, we will calculate intraclass correlations (ICCs) to observe the similarity of Neuroscan and EPOC^X^ waveforms, their peak amplitude and latency measures comparing the size and timing of each ERP peak.

Quantitative electroencephalography (qEEG) will be also analyzed during resting state. Signals will be processed according to the frequency range: delta (1–4 Hz), theta (4–8 Hz), slow alpha (8–10 Hz), fast alpha (10–13.5 Hz), and beta (13.5–30 Hz). Resting EEG recordings were recorded after 3 min with the participants’ eyes closed. For the analysis, we will select more than 2 min of artifact-free EEG readings from the three-minute recordings. As for specific waveforms, artifacts will be removed, using independent component analysis (ICA). EEG data for each subject will be analyzed using the short-time Fourier transform after removal of outliers that will be out of the spectral value distribution of each frequency band at the significance level of 0.05. The absolute powers will be then averaged at each bin and frequency band. For comparison analysis, Z-scores will be generated for each participant’s score.

### Data and safety monitoring committee

All data collected from participants will be recorded and secured on to physical- and cloud-based environment, certified for medical data. A separate secure file will contain contact information but has no personal data or protected health information that is collected as part of the study. Data will be collected at these intervals: only the research assistants/associates who have completed human research training will have access to the individually identifiable information about the participant and his/her family. Data will be collected by trained research assistants who have completed on-line training in human subjects’ research, HIPAA, and research integrity, training at each site on research data management and confidentiality. No personally identifying information will be coded on questionnaires, interviews, or other scoring forms, to protect confidentiality. Unique subject identification numbers are assigned to each participant. Only the Principal Investigator and key personnel who are HIPAA-trained have access to the file that links names with subject numbers, which will be stored separately from the de-identified data. All data will be stored in locked file cabinets in a locked office (paper/pencil files) and on password-protected computers located behind a secure and maintained firewall. Moreover, data will be collected specifically for this proposed research project.

All data will be anonymized for publication. Only researchers affiliated with the study will have access to participant data.

### Statistical analysis

Databasing, statistical software, and implementation of analyses will be programmed in the SAS Version 9.4, implemented along with plotting and graphing options. EEG analyses (e.g., mixed effects models) will be implemented at the Region of Interest (ROI) as well as the whole brain level.

#### Descriptive statistics

The following descriptive statistics will be presented:

▪ For quantitative variables: number of non-missing values, number of missing values, mean, standard deviation, minimum, maximum; median and first and third quartiles.
▪ For categorical variables: number of non-missing values, number of missing values and percentage. Unless otherwise specified, percentages will be calculated using the number of non-missing values as the denominator (i.e., not including missing values). Where appropriate, confidence intervals for proportions will be calculated using the adjusted Wilson score method (64).

#### Reference interval

There are several methods for constructing a reference interval for psychometric measures based on age and gender, all of which require a larger number of subjects the higher the precision desired. The minimum number of observations required per group has been set at 120 and reaffirmed in the National Committee for Clinical Laboratory Standards guidelines (65). For a 95% confidence interval around each percentile at the ends of the reference interval, this number increases to 153, and to 199 for a 99% confidence interval.

We will thus examine performance on neuropsychological tests, questionnaires, and academic assessments by:

▪ A univariate ANOVA to test for main effects and moderating effect of a qualitative variable.
▪ Linear multiple regressions and conditional analysis to test the moderating effect of a quantitative variable.
▪ And multiple linear regressions to test a mediating effect.

Machine- and deep-learning approaches will be used also to map the multi-dimensional of the cognitive, emotional, and behavioral measures. Unsupervised machine learning approaches will be to identify groups of children across dimensions and define transdiagnostic profile according to common cognitive, emotional, or behavioral profiles. Such methods will include class-based analyses (e.g., latent class or cluster analyses) and clustering algorithms.

#### Sampling

We will account for variations across population and strive to identify the broad range of social and environmental influences on brain function and development. Such an approach may ultimately reveal a much more complex tapestry of etiological mechanisms than are typically derived from averaging over a relatively homogenous sample. Based on our own experience and previous sampling frame to access a representative sample of the larger heterogeneous population of children (66–69) and using three broad dimensions of phonological processing abilities, executive skills and processing speed (70) with latent factor analysis (71), we estimated that the final sample size is 400 children (half boys and half girls) for power analysis of 0.8, a confidence level of 0.05, and a type I error to detect medium to small effects over the study’s duration (72) Because we except a 20% attrition rate at each critical step: the enrollment process till the data collection completion, we have estimated that 786 participants (Figure 1) will be needed to provide a comprehensive understanding of changes through the test batteries assessing numerous factors impacting brain functioning.

#### Hypothesis testing framework

Hypotheses will be tested at the α = 0.05 level after adjustment for multiple tests. Significant statistics only will be converted to indices of effect size and their associated confidence interval. The primary analytical framework will be nested linear mixed models with random effects (LMEs), and Cox proportional hazards models to handle quantitative predictor variables and categorical variables.

For LMEs, the primary effects of interest will generally be the interaction between independent variables and time (e.g., the visit number). Analyses will be performed with multiple levels of nesting (e.g., site, devices) that are not of intrinsic interest, as well as nesting within participants over time, allowing all data to be used for wide analyses. Age and gender will be specified as co-variables and others such as demographic and other background variables will be specified upon scientific and empirical considerations.

#### AI modeling

Our assessment tool is based on dynamic data modeling that includes the temporal aspect of multimodal signals in relation to the target variables, i.e., the behavioral trajectories of the children during the play phase. The interest of the dynamic approach is the number of samples processed per observation and per run, which increases the number of learning data.

This approach allows to analyze more signals in a single assessment and to synchronize them to obtain finer behavioral profiles that are sensitive to the inherent variability of individual performances and abilities than the classical approach where for each run all signals are summarized to a single observation, and consequently there will be few learning observations (one label per run). Recurrent neural networks and transformers have been chosen as prediction models for time series.

The prediction AI model consists of three main steps:

▪ The first one is to merge the features from the multiple signals, and structure them as multivariate time series ready to be used by machine learning models, since the prediction approach discussed is dynamic and will consider the dynamic aspect of the data. High-level feature extraction will be validated by expertise of psychologists and physicians. This choice is mainly for the explicability of the outputs of the system since it allows to detect the most relevant features and to find the causal relationships between them the target variables and the input features. This part can include already trained AI models. For example, the use of already trained deep learning networks for the detection of face and eye points, head position, etc., … This extraction also includes domain expertise. The latter is very important in this domain, i.e., high-level feature extraction from multimodal signals, as it is used to design what to extract in such a way that it is interpretable and effective for prediction based on human experience.
▪ A second step of variable selection will be applied to reduce the set of input variables and to identify the most relevant features. This step will also be useful for the output of the prediction module to detect causal relationships between high-level behavioral characteristics and the variables to be predicted, for example, children’s attention scores. On a technical level, the methods applied in this stage are part of the “feature selection” methods and can be supervised or unsupervised machine learning methods. In the learning phase, several methods can be tested to find the best one for each attention score.
▪ The third step is summarized in the prediction module which consists in applying prediction models with a sequence-to-label strategy, since the novelty of this tool will be to predict the scores of cognitive, metacognitive and behavioral functions in a dynamic way, i.e., to predict an attention score at an instant of time according to the past temporal sequences of the features extracted in the second step of the prediction module. The length of the sequences will be studied in more detail soon depending on the size of the available behavioral signals and will be adjusted in such a way as to improve the predictions. In this context, the classifiers used will be recurrent neural networks, for example the LSTM (Long Short-Term Memory) network, and transformers. A second feature can be easily added to the prediction tool which consists in classifying the children (NDD/neurotypical) starting only from the high-level features extracted from the raw behavior signals, without the need for labels (attention scores). In this case, unsupervised learning models of the “clustering” type are applied.

### Safety and adverse event assessment and monitoring

The promoter must warrant independent quality assurance (QA) audits of study processes, if deemed appropriate. The auditors/inspectors will have the right to inspect the study at any time during and/or after the completion of the study and will be granted access to source documents.

Security and data monitoring will be performed to ensure and maintain the scientific integrity of this project and protect the safety of our participants. Safety monitoring will involve the review of cumulative outcome data for groups of participants to determine if any of the procedures performed should be modified or stopped. Ongoing and close monitoring of participant safety will include prompt reporting of safety data (i.e., adverse/serious events) to the appropriate authorities with oversight responsibility and to the R&D project manager within 48 h of project personnel becoming aware of the incident. A summary of safety measures will be provided as part of the annual progress report, which resume the written report required by the competent authorities. In addition, participants will be informed of any significant new findings during the study (e.g., other potential risks) that may affect their willingness to continue participating in the study.

Although severe risks are minimal in this study, review of the data and procedures may result in early termination of the study, modification of the protocol, or changes in the data collection plan. If the protocol or data collection plans need to be modified after data review, the appropriate authorities will be notified, and the amendment will be approved prior to implementation of the study modification. Protocol deviation or non-compliance with the protocol of this study may be the fault of the participant, investigator, or study personnel. In the event of a deviation, corrective actions should be developed by the site and implemented promptly.

The data entry system includes password protection and internal quality controls, such as automatic interval checks, to identify data that appear inconsistent, incomplete, or inaccurate. Data will be entered directly from the source documents by an authorized person and changes to data will be made by an authorized person. All source documents must be completed in a neat and legible manner to ensure accurate interpretation of the data. Data recorded from the source documents must be consistent with the data recorded on the source documents. The investigator is responsible for the management and accuracy of the information contained.

## Discussion

We have described EPIDIA4Kids, a study protocol to evaluate how multimodality biometry relying on digital epidemiology can give insight into brain functioning in children aged 7 to 12 years. While they are playing psychometric games on an unmodified tablet, multi-stream data will be collected from 400 children volunteers independently of NDD diagnosis.

Multivariate linear models for brain functioning scores will be obtained by using supervised machine/deep-learning and cross-validation. Those models will include variables from the collected biometric data such as emotion estimates, eye-tracking features, digit-trajectories, correction rates and response time during game’s performances. Each brain functioning scores estimated by each biometric model will be agreed with each score of one or some questionnaires. This multimodality approach (73) reflects the recent advances in both quantity and quality of data available since Information & Communication Technology (ICT) and Internet of Things (IoT) brings high potential for improved accuracy at cost-efficiency (24, 28). Machine learning and AI applied to NDDs may open valuable route to examine heterogenous symptoms in pediatric population and at individual level, by integrating multimodality dimensions in prediction models, such as social, environmental, and structural determinants (74–76). Furthermore, the models can handle for missing data and larger numbers of interactive predictors. The so-called digital epidemiology could thus provide support more accurate and objective diagnostic and therapeutic decision making to health practitioners in their practice.

However, while we aim at building comprehensive datasets to create meaningful metrics of cognition, emotions and behaviors based digital epidemiology, we will carefully review data quality to lower response bias (e.g., youth’s illectronism, intra-and inter-examiner variance) by, for instance, minimizing heterogeneity of hardware implementation, and AI-related bias (e.g., over/underfitting). Furthermore, these *de novo* biometrics will require mix adjustment to ensure that observed differences in outcomes are relevant to the actual clinical categorization.

Categorical diagnoses are the existing taxonomies for NDDs. Firstly, they fail to capture a holistic view of the learning, behavior, or social functioning in children. Secondly, their current thresholds are often dependent of various scales enable to identify subtle disabilities of individual although their life are significantly altered [(15), for review]. They minimize then the high variability within symptoms, meaning that children having the same diagnostic label can be impacted differently in nature and severity of their symptoms (30, 77, 78). Lastly, symptoms overlap across categorical diagnoses, leading to unreliable intervention choice (79, 80). Transdiagnostic appears then as an alternative to improve individual heterogeneity along with the symptom network theory models of psychopathology (81), and better address patient specificity in diagnosis and therapeutic recommendations by using dimensions of discrete categories (82–84) as previously endorsed by the NIMH Research Domains Framework (RDoC) (85, 86). Transdiagnostic approach applied to NDDs (15, 70, 82, 87–89) may reflect brain functioning and give insight into mechanism of neurodevelopment.

Altogether evidence have supported that the XAI4Kids® system combining biometric measures and transdiagnostic approaches may provide compelling alternatives in capturing the heterogeneity of NDDs in the population at large scale and more feasible and acceptable in real-life practice than the current standards.

## Data availability statement

The datasets generated for this study will not be made publicly available because it was not included in the ethics approval to submit the datasets.

## Ethics statement

The studies involving human participants were reviewed and approved by Comité de Protection des Personnes Sud-Est II approved the protocol under the reference 2022-A00766-37. Written informed consent to participate in this study will be provided by the participants’ legal guardian/next of kin.

## Author contributions

VD conceived and designed the study, drafted the grant and the protocol manuscript, will organize, and supervise study implementation, management, and staff training, and will act as study project manager. TM and AH provided statistical expertise in the study design and participated in study implementation. HC and PS provided expertise on study methodology and advises on management. HC provided expertise in clinical neurodevelopment. PS provided expertise in epidemiology in public health. TM will act as clinical research assistant, monitoring recruitment and data collection, and liaising with recruitment sites. All authors contributed to the article and approved the submitted version.

## Funding

The study was funded partially by the bank of innovation (grant: DOS0180634/00) to O-Kidia. The funding institution will have no role in study design, data collection, data analysis and interpretation, writing the manuscripts or the decision to submit for publication.

## Conflict of interest

TM and AH are employees of O-Kidia. VD has stock and ownership interest in O-Kidia. As part of the study, VD, TM, and AH report being Principal Investigator/Investigator of the present study under the supervision of the Comité de Protection des Personnes Sud-Est II (CPP 69).

The remaining authors declare that the research was conducted in the absence of any commercial or financial relationships that could be construed as a potential conflict of interest.

## Publisher’s note

All claims expressed in this article are solely those of the authors and do not necessarily represent those of their affiliated organizations, or those of the publisher, the editors and the reviewers. Any product that may be evaluated in this article, or claim that may be made by its manufacturer, is not guaranteed or endorsed by the publisher.

## Footnotes

^1^ unity3d.com
